# Comparation of quality of life in Chinese patients undergoing leadless versus conventional pacemaker implantation

**DOI:** 10.1002/clc.23939

**Published:** 2022-11-02

**Authors:** Miao Yu, Yue P. Li, Dong M. Shi, Yu J. Zhou

**Affiliations:** ^1^ Department of Cardiology, Beijing Anzhen Hospital, Capital Medical University, Beijing Institute of Heart Lung and Blood Vessel Disease, Beijing Key Laboratory of Precision Medicine of Coronary Atherosclerotic Disease, Clinical Center for Coronary Heart Disease Capital Medical University Beijing China

**Keywords:** conventional pacemakers, leadless pacemaker, Pacemaker, QoL, SF‐36

## Abstract

**Background:**

Leadless pacemakers are widely used, but the quality of life assessment of patients with leadless pacemakers is still unclear.

**Hypothesis:**

Assume that leadless pacemakers can improve the patients' quality of life.

**Methods:**

Total of 119 patients who received pacemaker implantation at Beijing Anzhen Hospital from January 2020 to March 2022 were selected, including 35 leadless pacemakers and 84 conventional pacemakers. The SF‐36 questionnaire was used to evaluate quality of life at baseline, 1 month and 3 months after surgery. We also used a questionnaire consisted of 4 specific questions related to the implant procedure to assess the surgery.

**Results:**

There were no differences in baseline characteristics between the two groups, except for age and oral anticoagulant treatment. There was no difference in baseline SF‐36 scores. At the 3‐month follow‐up, patients in leadless pacemakers group were significantly better at physical function (63.63 vs 47.50, *p* = .000), role physical (60.20 vs 40.23, *p* = .000), bodily pain (65.57 vs. 61.69, *p* = .042), physical component summary (61.25 vs. 50.57, *p* = .000), vitality (56.26 vs 49.57, *p* = .001), social function (80.14 vs 74.70, *p* = .004), role emotional (76.14 vs. 71.42, *p* = .015), mental health (75.46 vs. 68.18, *p* = .000), mental component summary (72.00 vs. 65.97, *p* = .000), even after adjusting for clinical baseline and SF‐36 baseline. Pacemaker‐related discomfort and mobility limitations were significantly reduced in leadless pacemakers group.

**Conclusions:**

Leadless pacemakers is associated with better quality of life with less activity limitations due to surgical discomfort and less emotional distress. However, current use of leadless pacemakers in China is limited due to the high cost.

## 1 INTRODUCTION

Cardiac conduction system diseases are common diseases with high morbidity, every year thousands of patients are treated with pacemakers (PM).^1^ Since the first permanent pacemaker was implanted, the pacing system has gradually improved as technology has developed. Whether the size, battery life, or pacemaker programming, are all constantly improving. Leadless pacemakers (L‐PM) do not need a subcutaneous pocket for the pulse generator and permanent transvenous leads, thus reducing the incidence of surgical complications, are gradually replacing the implantation of conventional pacemakers (C‐PM).^2^, ^3^

Some studies have confirmed that C‐PM can prolong the survival of patients with bradyarrhythmias, and simultaneously improve the quality of life (QoL).^4^, ^5^, ^6^, ^7^ Though in such patients L‐PM can also provide a safe and effective treatment,^8^, ^9^ the QoL status of patients with L‐PM is still unclear. The aim of this study was to evaluate the difference in QoL between patients with C‐PM and patients with L‐PM, and to provide a basis for the selection of patient treatment strategies.

## 2 METHODS

### 2.1 Study population

This is a single‐center observational study. Total of 119 patients who received pacemaker implantation at the 12th ward of Beijing Anzhen Hospital from January 2020 to March 2022 were included in this study. The principles of surgical selection are based on clinical diagnosis, patient preference, and operator experience. The main inclusion criteria included: (1) The patients had the indication of pacemaker implantation; (2) The patients had no cognitive disorder, and signed informed consent to complete the SF‐36 quality of life questionnaire. The main exclusion criteria included: (1) The patients had surgical intervention or invasive treatment 3 months before the pacemaker implantation; (2) The patients had other indications for surgical intervention at the time of pacemaker implantation. Total of 35 patients with L‐PM and 84 patients with C‐PM were included in this study. All patients were fill out the SF‐36 questionnaire before the pacemaker implantation, also at the follow‐up time at 1 month and 3 months after implantation. Complications include pneumothorax, pocket hematoma, electrode dislocation, and hematoma, hemorrhage, pseudoaneurysm, arteriovenous fistula in femoral vein puncture site.

### 2.2 QoL

QoL was measured by using the SF‐36 Quality of Life Questionnaire. SF‐36 is consists of 36 questions, widely used in clinical work,^10^, ^11^, ^12^, ^13^ including eight dimensions for evaluating health‐related quality of life (HRQOL). Divided into two categories: physical health and mental health, physical function (PF), role physical (RP), bodily pain (BP), general health (GH), vitality (VT), social function (SF), role emotional (RE), and mental health (MH). The scales most associated with physical health were physical function, role physical, bodily pain, general health, while vitality, social function, role emotional, and mental health were most closely associated with mental health. The scale is scored from 0 to 100. Additionally, the SF‐36 can be further divided into two composite scores, the physical component summary (PCS) and the mental component summary (MCS).

### 2.3 Additional questionnaire

In addition to the general questionnaire SF‐36, at follow‐up time, all patients completed an additional questionnaire containing of four specific questions related to pacemaker implantation, as follows:

(1) Have you felt discomfort in your surgical area (chest/groin)?
  − Yes ‐No

(2) Have you been restricted in your daily activities by discomfort in the region of the intervention?
  − Yes ‐No

(3) Have you been concerned about your heart condition and general health since your pacemaker implantation?
  − Yes ‐No

(4) Have you been depressed since your pacemaker implantation?
  − Yes ‐No

All the patients completed a SF‐36 questionnaire before the pacemaker implantation as the baseline. At 1 and 3 months after implantation, the SF‐36 questionnaire and the additional questionnaires (4 questions) were completed.

### 2.4 Statistical analysis

Quantitative variables are expressed as means and standard deviations, and qualitative variables are expressed as frequencies and percentages. Baseline characteristics of the study population were compared using chi‐square or Fisher's exact tests for categorical variables and two‐sample *t*‐tests for continuous variables. The *t*‐test was used to compare the SF‐36 scale, and the chi‐square test was used to compare the additional questionnaire. Mean change in SF‐36 from baseline to follow‐up was expressed as mean and standard error of the mean. Multivariate linear regression analysis was used to assess the effect of pacemaker implantation (C‐PM and L‐PM) on change in QoL at 3 months from baseline. Input variables were baseline SF‐36 scores and baseline characteristics (age) that differed between the two groups. Linear regression analysis was performed using SPSS v.26, and R software was used for other statistical analyses. *p* < .05 considered the difference to be statistically significant.

## 3 RESULTS

### 3.1 Patient sample characteristics and baseline QoL results

A total of 119 patients (35 L‐PM, 84C‐PM) were included in this study. Table 1 summarizes the baseline characteristics and QoL data of the two groups of patients. Seventy‐four male patients were enrolled, accounting for 62.2% of the total patients. The mean age of patients was 70.34 ± 9.79 years old. The two groups were statistically different in age (76.17 ± 7.92 vs. 67.92 ± 9.49, *p* = .004) and oral anticoagulant treatment (60% vs. 35.7%, *p* = .015). This difference was considered to be related to the fact that the patients of C‐PM group did not distinguish with a single‐chamber pacemaker or a dual‐chamber pacemaker. After implantation of the pacemaker, the patients in the C‐PM group need to have bed rest and immobilization for 24 h, and the upper limbs on the affected side could not be raised and restrict in activities within 3 months; the patients in the L‐PM group stayed in bed for 6 h after the operation, and no limbs restricted. The incidence of surgical complications in the L‐PM group was significantly lower than C‐PM group (0 vs. 18, *p* = .003). There was no significant difference between the two groups in the preoperative SF‐36 score (baseline QoL).

**Table 1:** Baseline characteristics and baseline SF‐36

	L‐PM group (*n* = 35)	C‐PM group (*n* = 84)	*p* value
Clinical characteristic			
Age, mean ± SD	76.17 ± 7.92	67.92 ± 9.49	.000
Male, *n* (%)	23 (65.7)	51 (60.7)	.608
Hypertension, *n* (%)	21 (60.0)	58 (69.0)	.341
Diabetes, *n* (%)	13 (37.1)	34 (40.5)	.735
Structural heart disease, *n* (%)	6 (17.1)	29 (34.5)	.058
Renal insufficiency, *n* (%)	7 (20.0)	10 (11.9)	.250
Coronary heart disease, *n* (%)	9 (25.7)	22 (26.2)	.957
Heart failure class III or IV, *n* (%)	4 (11.4)	7 (8.3)	.595
Cerebrovascular disease, *n* (%)	6 (17.1)	10 (11.9)	.445
Oral anticoagulant therapy	21 (60.0)	30 (35.7)	.015
Oral antiplatelet drug therapy	11 (31.4)	25 (29.8)	.857
Heart rhythm			
Atrial fibrillation	20 (57.1)	34 (40.5)	.096
Others, *n* (%)	15 (42.9)	50 (59.5)	.096
Surgery‐related complications *n* (%)	0 (0)	18 (21.4)	.003
QoL data (SF‐36)			
PF	41.83 ± 10.76	41.21 ± 5.85	.752
RP	27.51 ± 10.46	30.69 ± 12.69	.194
BP	48.40 ± 9.58	48.98 ± 8.48	.746
GH	44.83 ± 10.44	44.52 ± 6.60	.874
VT	39.94 ± 9.88	41.52 ± 8.66	.386
SF	61.77 ± 15.04	65.00 ± 11.00	.195
RE	57.23 ± 8.72	59.44 ± 7.74	.174
MH	55.94 ± 12.76	59.55 ± 8.00	.128
PCS	40.64 ± 7.17	41.35 ± 6.05	.583
MCS	53.72 ± 8.58	56.38 ± 5.83	.100

Abbreviations: BP, bodily pain; GH, general health; MCS, mental component summary; MH, mental health; PCS, physical component summary; PF, physical function; RE, role emotional; RP, role physical; SF, social function; VT, vitality.

### 3.2 QoL at follow‐up

All 119 patients completed the SF‐36 questionnaire and additional questionnaire at 1 month ± 7 days and 3 months ± 7 days after implantation.

The SF‐36 scores at the 1‐ and 3‐month follow‐ups are shown in Table 2. There were statistically significant differences in PF, RP, BP, GH, VT, RE, PCS, and MCS between the two groups in 1‐month, and significant differences in PF, RP, BP, VT, SF, RE, MH, PCS, and MCS in 3‐month. The results in the L‐PM group were significantly better than the C‐PM group. After adjusted for clinical baseline and SF‐36 baseline, the linear regression analysis results shown that the L‐PM group was significantly better than the C‐PM group in terms of PF, RP, GH, VT, SF, RE, and MH at 1‐month follow‐up, and the differences in PF, RP, VT, SF, RE, MH were still statistically significant at 3‐month (Table 3).

**Table 2:** Mean SF‐36 scores and additional questionnaire during 1‐ and 3‐month follow up

QoL data (SF‐36)	L‐PM group (*n* = 35)	C‐PM group (*n* = 84)	*p* value
1‐Month follow up			
PF	56.51 ± 13.03	42.9 ± 8.33	.000
RP	52.63 ± 14.61	24.80 ± 8.33	.000
BP	59.91 ± 12.01	54.50 ± 13.47	.042
GH	55.11 ± 10.97	45.48 ± 7.91	.000
VT	54.26 ± 13.00	45.29 ± 8.73	.000
SF	74.20 ± 14.65	69.42 ± 11.20	.055
RE	71.06 ± 11.20	62.15 ± 9.18	.000
MH	70.97 ± 10.75	67.57 ± 9.61	.092
PCS	56.05 ± 10.15	41.92 ± 5.87	.000
MCS	67.62 ± 9.45	61.12 ± 6.72	.000
Additional Questionnaire (Yes/No, *n* [%])			
Felt discomfort in surgical area (chest/groin)	7 (20.0)	40 (47.6)	.005
Restricted daily activities by discomfort in the region of the intervention	5 (14.3)	46 (54.7)	.000
Concerned about your heart condition and general health	7 (20.0)	54 (64.3)	.000
Felt depressed	7 (20.0)	24 (28.6)	.332
3‐Month follow up			
PF	63.63 ± 9.97	47.50 ± 7.94	.000
RP	60.20 ± 10.73	40.23 ± 9.43	.000
BP	65.57 ± 9.52	61.69 ± 9.35	.042
GH	55.60 ± 12.56	52.37 ± 9.13	.175
VT	56.26 ± 10.84	49.57 ± 9.17	.001
SF	80.14 ± 10.83	74.70 ± 8.64	.004
RE	76.14 ± 10.28	71.42 ± 6.34	.015
MH	75.46 ± 10.27	68.18 ± 9.99	.000
PCS	61.25 ± 8.17	50.57 ± 5.98	.000
MCS	72.00 ± 6.42	65.97 ± 5.65	.000
Additional Questionnaire (Yes/No, *n* [%])			
Felt discomfort in surgical area (chest/groin)	4 (11.4)	30 (35.7)	.008
Restricted daily activities by discomfort in the region of the intervention	4 (11.4)	32 (38.1)	.004
Concerned about your heart condition and general health	8 (22.9)	37 (44.0)	.030
Felt depressed	4 (11.4)	20 (23.8)	.125

Abbreviations: BP, bodily pain; GH, general health; MCS, mental component summary; MH, mental health; PCS, physical component summary; PF, physical function; RE, role emotional; RP, role physical; SF, social function; VT, vitality.

**Table 3:** 1‐ and 3‐month linear model regression results

		Univariate			Model 1			Model 2		
		Coefficient B	95% CI	*p* value	Coefficient B	95% CI	*p* value	Coefficient B	95% CI	*p* value
1‐month score	PF	−0.553	−17.565, −9.655	.000	−15.614	−19.775, −11.453	.000	−14.224	−18.316, −10.131	.000
	RP	−0.772	−32.032, −23.629	.000	−29.930	−34.442, −25.417	.000	−29.345	−33.361, −25.329	.000
	BP	−5.414	−10.618, −0.210	.042	−6.271	−11.920, −0.623	.030	−5.271	−10.551, 0.008	.050
	GH	−9.638	−13.202, −6.074	.000	−10.978	−14.777, −7.180	.000	−10.861	−14.689, −7.033	.000
	VT	−8.971	−13.020, −4.923	.000	−11.031	−15.386, −6.677	.000	−11.032	−15.242, −6.823	.000
	SF	−4.783	−9.680, 0.114	.055	−7.447	−12.668, −2.227	.006	−7.182	−10.751, −3.614	.000
	RE	−8.902	−12.812, −4.993	.000	−11.232	−15.401, −7.063	.000	−11.262	−15.062, −7.463	.000
	MH	−3.400	−7.367, 0.567	.092	−5.612	−9.858, −1.366	.010	−6.297	−10.289, −2.304	.002
3‐month score	PF	−16.129	−19.545, −12.712	.000	−17.817	−21.486, −14.147	.000	−17.214	−20.945, −13.483	.000
	RP	−19.974	−23.889, −16.059	.000	−21.396	−25.243, −17.150	.000	−20.805	−24.503, −17.108	.000
	BP	−3.881	−7.625, −0.137	.042	−4.444	−8.556, −0.332	.034	−4.048	−8.108, 0.011	.051
	GH	−3.231	−7.314, 0.852	.120	−4.017	−8.479, 0.446	.077	−3.508	−7.911, 0.895	.117
	VT	−6.686	−10.544, −2.827	.000	−7.374	−11.582, −3.167	.001	−7.375	−11.518, −3.232	.001
	SF	−5.440	−9.157, −1.724	.004	−6.703	−10.754, −2.652	.001	−6.593	−10.335, −2.851	.001
	RE	−4.726	−7.784, −1.668	.003	−6.356	−9.625, −3.067	.000	−6.361	−9.541, −3.180	.000
	MH	−7.279	−11.292, −3.265	.000	−8.803	−13.167, −4.440	.000	−9.138	−13.472, −4.805	.000

*Note*: Model 1: adjusted for age and Oral anticoagulant therapy. Model 2: adjusted for clinical baseline and SF‐36 baseline.

Abbreviations: BP, bodily pain; CI. confidence interval; GH, general health; MCS, mental component summary; MH, mental health; PCS, physical component summary; PF, physical function; RE, role emotional; RP, role physical; SF, social function; VT, vitality.

Table 2 also summarized the results of the additional questionnaire related to pacemaker implantation. It showed that the C‐PM group had higher rates of discomfort in the surgical area, disturbance of daily activities due to discomfort in the surgical area, and concerns about cardiac conditions and general health. At 1‐ and 3‐month follow‐up results, three numerical differences were statistically significant.

### 3.3 Change of QoL from baseline to follow‐up

**Figure 1:**
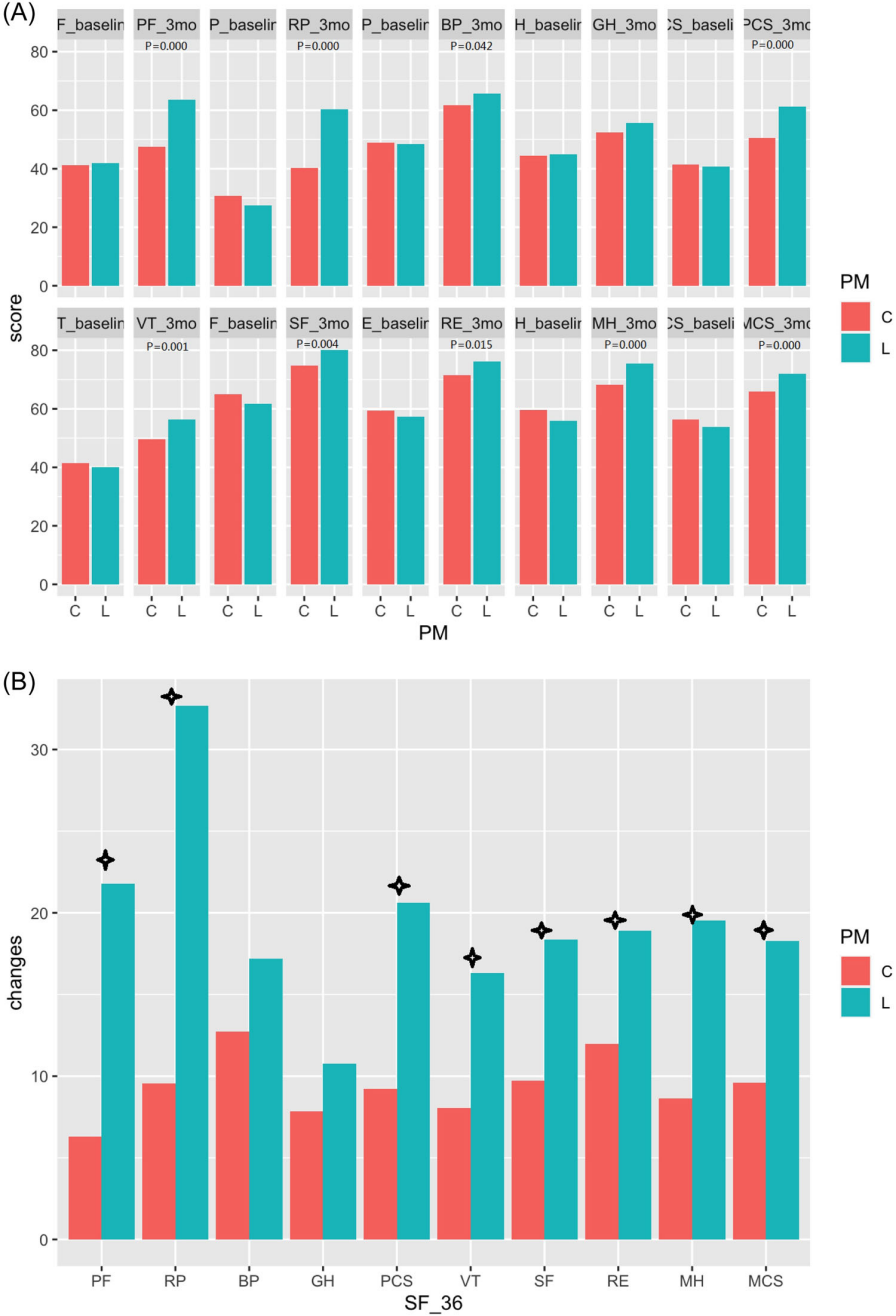
Include A and B. (A) SF‐36 scores at baseline and 3‐month after the implantation. (B) Mean changes from baseline to 3‐month follow‐up. C‐PM, conventional pacemakers; L‐PM, leadless pacemakers; *p* < .05 vs. control. BP, bodily pain; GH, general health; MCS, mental component summary; MH, mental health; PCS, physical component summary; PF, physical function; RE, role emotional; RP, role physical; SF, social function; VT, vitality.

From baseline to 3‐month follow‐up, all SF‐36 scores increased gradually, and all items were statistically different in the two groups compared with baseline, but the numerical changes were more pronounced in the L‐PM group. There were statistically significant differences in the changes of PF, RP, PCS, VT, SF, RE, MH, and MCS between the two groups (Figure 1 and Table 4).

**Table 4:** Mean changes from baseline to follow‐up

SF‐36	L‐PM group	C‐PM group	*p*	*p* *	*p* **
ΔPF	21.80 ± 14.15	6.29 ± 8.11	.000	.000	.000
ΔRP	32.69 ± 13.09	9.54 ± 9.95	.000	.000	.000
ΔBP	17.17 ± 12.06	12.71 ± 11.26	.066	.000	.000
ΔGH	10.77 ± 15.34	7.85 ± 9.54	.301	.000	.000
ΔPCS	20.61 ± 9.67	9.21 ± 5.23	.000	.000	.000
ΔVT	16.31 ± 12.90	8.05 ± 11.33	.002	.000	.000
ΔSF	18.37 ± 14.11	9.70 ± 11.00	.002	.000	.000
ΔRE	18.91 ± 11.53	11.98 ± 8.15	.002	.000	.000
ΔMH	19.51 ± 14.46	8.63 ± 11.65	.000	.000	.000
ΔMCS	18.28 ± 8.10	9.59 ± 6.61	.000	.000	.000

*Note*: SF‐36 data are presented as mean ± standard error. *p*, L‐PM vs. C‐PM; p*, L‐PM vs. L‐PM; p**, C‐PM vs. C‐PM.

## 4 DISCUSSION

This study shown that patients in the L‐PM group had more significant improvements in physical health, higher scores in PF and RP, and less discomfort in surgical area and restricted mobility due to discomfort. Consider this result is relation to the improvements of devices, as well as differences in implantation techniques. L‐PM is to implant the pacemaker into the right ventricle through a catheter delivery system via the femoral vein; while C‐PM implantation, venous access (most commonly the subclavian vein, axillary vein, or cephalic vein) is required to lead into the right ventricle, and the pulse generator is usually placed in the pocket in the subclavian region. Therefore, after C‐PM implantation, the patient's pocket should be compressed for 24 h, and the affected upper limb should be completely immobilized for 24 h. Within 3 months after the operation, it is recommended that the affected upper limb be relatively immobilized to avoid lifting and extracting heavy objects. The aim is to avoid complications (dislocation of electrodes, pocket injury).^14^ However, after L‐PM implantation, the patient only needs to press the femoral vein of the affected side for 6 h, and there is no subsequent braking requirement. These all cause discomfort and mood swings in patients with C‐PM, and thus may explain the differences in physical health that we found at 1‐ and 3‐month follow‐up. This is also in line with the results of the additional questionnaire, for such discomfort and mood will cause patients to reduce physical activity on their own. In contrast, patients in the L‐PM group experienced less discomfort in the surgical field and a reduction in daily activities after implantation, which was positively correlated with the improved physical health outcomes found in the SF‐36 questionnaire.

Furthermore, we found the characteristics of changes in SF36 from baseline to 3‐month follow‐up in the C‐PM and L‐PM groups. The data showed a general improvement in QoL from baseline to 3 months regardless of the pacemaker implanted, and there was a statistically significant difference between the two groups in the 3‐month score compared with baseline. This is consistent with previous research on QoL after C‐PM implantation, which showed a general improvement in both physical and mental health of patients.^6^, ^7^, ^15^, ^16^, ^17^, ^18^, ^19^, ^20^ A recent study by Fleur et al.^21^ showed a significant improvement in patients' QoL and high patient satisfaction after L‐PM implantation. In our study shows that although the implantation of pacemakers can improve QoL in both groups, the numerical changes in the L‐PM group are more obvious, and there are significant differences in PF, RP, PCS, MH, and MCS between the two groups are more obvious. Furthermore, L‐PM implantation was significantly better than C‐PM in terms of local discomfort, restricted mobility, and the mood swings.

There are some limitations in this study: the sample size of this study is small, because the price difference between L‐PM and C‐PM in China is huge, and most patients cannot afford the high price of L‐PM. Moreover, this study is a single‐center study, which may cause statistical bias. This study used the SF‐36 general questionnaire, which provides an overview of health status and is one of the most commonly used questionnaires for clinical outcomes. Meanwhile, we designed an additional questionnaire for patients with pacemakers to minimize the general questionnaire limitations when evaluating specific conditional problems. Although the additional questionnaire was designed based on clinical experience, it has not been confirmed.

In this study, all comparisons between the C‐PM and L‐PM groups were performed in non‐randomized studies. Although there were no differences in sample characteristics with the exception of age and oral anticoagulant, and no differences in baseline quality of life data, further randomized controlled studies are needed to confirm our results.

## CONCLUSION

L‐PM is associated with better QoL, and patients who received L‐PM had fewer surgery‐related complications, less activity limitations due to surgical discomfort, and less emotional distress. However, current use of L‐PM in China is limited due to the high cost.

## CONFLICT OF INTEREST

The authors declare no conflict of interest.

## Data Availability

The data that support the findings of this study are available from the corresponding author upon reasonable request.
